# Review of "*Keys to the Trematoda. Vol. 3*" by Rodney A. Bray, David I. Gibson and Arlene Jones (eds.)

**DOI:** 10.1186/1756-3305-2-9

**Published:** 2009-01-29

**Authors:** Aneta Kostadinova

**Affiliations:** 1 Institute of Parasitology, Biology Centre of the Czech Academy of Sciences, Branišovská 31, 370 05 České Budějovice, Czech Republic; 2 Central Laboratory of General Ecology, Bulgarian Academy of Sciences, 2 Gagarin Street, 1113 Sofia, Bulgaria

## Review

The third and final volume of a series on the systematics and identification of the platyhelminth class Trematoda Rudolphi, 1808, perhaps one of the most important taxonomy endeavours of this century, is now available.

The volume includes five superfamilies of the Order Plagiorchiida (Gorgoderoidea Looss, 1899; Microphalloidea Ward, 1901; Monorchioidea Odhner, 1911; Opisthorchioidea Looss, 1899; and Plagiorchioidea Lühe, 1901) and the family Didymozoidae Monticelli, 1888 of the Order Strigeida which was omitted from Volume 1. As in the previous volumes, the core of the book (59 chapters covering 618 genera) provides taxonomic background which invariably represents top-class discussion based on extensive knowledge of the history of the group and of the literature, familial diagnoses, keys and diagnoses to the genera and subfamilies (where applicable). A further five chapters give short introductions and diagnoses of the superfamilies, and keys to families. Finally, the volume includes a linking key to all superfamilies of the Digenea with a reference to the volume of the series where treated in detail, and a separate chapter comprising diagnoses of 18 genera *incertae sedis *in the superfamily Plagiorchioidea (*sensu lato*), lists of digenean generic names (genera *inquirenda*, larval names and *nomina nuda*) not used in the three volumes of keys and a list of the new genera erected after the publication of the first two volumes of the series. The third volume thus provides completeness to this encyclopaedic work. The text is admirably referenced and remarkably free from typographical errors.

The novelty of this volume lies in the greater effort put towards reaching an agreement between morphological and molecular taxonomy of the Digenea. This, although leading to problems with key construction based on adult morphology alone, is a step towards a natural system of the plagiorchiidans and a sound basis for future molecular studies addressing phylogenetic relationships at the suprageneric level.

All chapters are written by a team of international experts, 18 leading scientists from Australia (D.P. Barton, D. Blair, T.H. Cribb, T.L. Miller and J. Pearson), Brazil (C.P. Santos), Czech Republic (T. Scholz), France (S. Deblock), India (R. Madhavi), Poland (T. Pojmañska), Russia (S.E. Pozdnyakov), UK (R.A. Bray, D.I. Gibson and A. Jones), and USA (R.A. Campbell, W.F. Font, J.M. Lotz and V.V. Tkach). As in the previous volumes a profound editorial effort has brought coherence to the book although some authors use additional taxonomic categories (supersubfamily, tribe) or diagrammatic representation of the genera. Another 'deviation' considered by some authors is a description of the characters used in diagnosing genera which I find very useful especially because it is associated with difficult families such as the Cryptogonimidae Ward, 1917, Microphallidae Ward, 1901 and Didymozoidae Monticelli, 1888.

The authors have presented novel concepts for the taxonomy of the groups covered often in conjunction with molecular evidence and all chapters are, therefore, original. A substantial effort has been made towards reappraisal of the generic diagnoses *via *re-examination of type and/or other representative species. Application of an additional 'originality' metric based on the proportion of the original illustrations reveals uneven distribution across the chapters apparently related to the availability of the type- and/or comparative material with the highest values of 'originality' for the taxa covered by S. Deblock (88%), J. Pearson (78%) and T. Scholz (72%) followed by V.V. Tkach (47%) and R.A. Bray (46%). The style of the illustrations differs but line drawings are clear in spite of the reduction (though some dicrocoeliid figures are too small) and aid in the portrayal of generic features.

This book represents the most important contribution to the systematics and identification of the taxa covered and, together with the previous two volumes, will serve as essential unique source of biodiversity information well into the 21st Century. The volume is essential for taxonomists and the comprehensive keys will be valuable for parasitologists, both experts and novices, who study the diversity of parasites of wild-life vertebrates. It also includes groups of veterinary and medical significance such as the Dicrocoeliidae and Opisthorchiidae thus having direct relevance to human and veterinary parasitologists. No doubt this long awaited volume will join the first two in the libraries of schools of biological, medical and veterinary sciences and parasitology research laboratories. I congratulate the editors and authors and recommend it highly.

## Competing interests

The author has contributed to a previous volume in this series.

